# Editorial: Microbial Fabrication of Nanomaterials and Their Applications

**DOI:** 10.3389/fchem.2021.739739

**Published:** 2021-07-23

**Authors:** Sougata Ghosh, Karishma R. Pardesi, Cristina Satriano, Avinash Manjula Basavanna

**Affiliations:** ^1^ Department of Microbiology, School of Science, RK University, Rajkot, India; ^2^ Department of Microbiology, Savitribai Phule Pune University, Pune, India; ^3^ Department of Chemical Sciences, University of Catania, Catania, Italy; ^4^ Wyss Institute for Biologically Inspired Engineering, Harvard University, Boston, MA, United States; ^5^ Department of Chemistry and Chemical Biology, Northeastern University, Boston, MA, United States; ^6^ Department of Nuclear Science and Engineering, Massachusetts Institute of Technology, Boston, MA, United States

**Keywords:** microbial factories, biosynthesis mechanism, bionanotechnology, nanomedicine, biosensor, sustainability, catalysis

The Research Topic entitled “Microbial Fabrication of Nanomaterials and Their Applications,” was dedicated to original research reports and reviews on novel nanoparticles synthesized by microbes and their metabolites for various applications, including pharmaceutics, food, agriculture, environment, and electronics.

Nanotechnology is a multidisciplinary field that deals with the fabrication of nanomaterials (NMs) with attractive physicochemical and opto-electronic properties. In nanotechnology, interest in biosynthesis of nanoparticles is increasing, particularly in the use of unicellular and subcellular supports. Indeed, environmentally benign green routes of nanoparticles synthesis have gained significant attraction compared to traditional physical and chemical processes, which may pose potential threat to the environment owing to, e.g., the involvement of hazardous agents. Bacteria-based biosynthesis methods offer the advantage of a significant room for improvement in the quality of the nanoparticles synthesized, in terms of stability as well as of size and shape homogeneity of the nanoparticles.

The articles published under this research topic cover microbially fabricated nanomaterials that are synthesized either intracellularly or extracellularly where reducing enzymes, functional groups of non-enzyme proteins or polysaccharides are involved in the synthesis and shape evolution.

Nadhe et al. reported bacteriogenic synthesis of gold nanoparticles (AuNPs) using *Acinetobacter* sp. cells. Interestingly the optimization studies reflected that rate of synthesis was dependent on several factors that include culture age, cell density, pH and metal salt concentration. The resulting monodispersed bacteriogenic AuNPs, having particle size of 15 ± 10 nm, were released by the bacterial cell in the supernatant at 37°C. The AuNPs possessed superior antioxidant activity.

Another very interesting study by Jain et al. published in this special issue reported an economical and eco-friendly synthesis of ZnONPs using a zinc-tolerant bacteria, *Serratia nematodiphila*. ZnONPs were synthesized by using 0.1 M zinc sulphate solution and bacterial culture. The size of the nanoparticles ranged from 15 to 30 nm with a mean diameter of 23.09 ± 4.23 nm. The bacteriogenic ZnONPs showed superior antimicrobial activity against phytopathogenic bacteria, *Xanthomonas oryzae* pv. oryzae and fungus *Alternaria* sp. ZnONPs also exhibited significant catalytic methyl orange dye degradation (90%) after 80 min. The above reports demonstrated the agricultural and environmental applications of biogenic nanoparticles.

The review article by Gupta and Shukla, showcased the nanotechnological applications of nanocellulose, which comprises of attractive properties such as biodegradability, biocompatibility, non-toxicity, and environmental-friendliness. Meena et al. reviewed the nanobiotechnological prospects of endophytic microflora. Apart from biogenic nanoparticles, the approaches for the synthesis and applications of magnesium oxide nanoparticles (MgO-NPs), copper nanoparticles (Cu-NPs), chitosan nanoparticles (CS-NPs), β-d-glucan nanoparticles (GNPs), and engineered nanoparticles were also included. Another review by Sachin and Karn presents a comprehensive account on diverse applications of microbially derived nanoformulations as a drug delivery and targeting agent.

Among all the known metal nanoparticles, platinum nanoparticles (PtNPs) are of significant interest to the scientific community due to their diverse biomedical applications, namely, imaging, implants, photothermal therapy and drug delivery. Bloch et al. presented a most comprehensive report on biogenic PtNPs synthesized by several bacteria such as *Acinetobacter calcoaceticus, Desulfovibrio alaskensis, Escherichia coli, Shewanella algae, Plectonema boryanum,* etc. along with their mechanism of synthesis. Further, they reported the applications of bacteriogenic PtNPs in treatment of pharmaceutical products, cancer prevention, catalysis, control of bacterial infections and oxidative stress management. Additionally, an overview of various types of nanoparticles such as, TiO_2_NPs, AgNPs, AuNPs, CuNPs, SeNPs, PdNPs, CdSNPs, ZnONPs, Fe_3_O_4_NPs, ZrO_2_NPs and many more nanostructures synthesized biologically using bacteria, fungi and algae are also covered in great depth in this research topic (Ghosh et al.).

Nag et al. have discussed potential alternatives to inhibit and eradicate bacterial biofilms using biogenic nanoparticles. They highlighted the promising applications of enzyme functionalized metallic nanoparticles, silver–platinum nanohybrids (AgPtNHs), nanowires, superparamagnetic iron oxide nanoparticles synthesized by both magnetotactic and non-magnetotactic bacteria for degradation of the exopolysaccharide matrix.

The content of this research topic can be summarized as microbe assisted fabrication of exotic nanostructures for biomedical, environmental, agricultural and food related applications. Further, the identification of the microbial metabolites responsible for shape evolution and size determination during the process of reduction and capping would certainly allow us to develop standardized biological processes using microbial biomolecules for synthesis of nanostructures with tunable shape and size for desired applications. Thus, this research topic on microbial factories has shed interesting insights into synthesis, mechanisms, properties and applications of microbially produced nanomaterials, and provides an exciting opportunity to look at the “small” to fix the bigger problems.

